# Photogenerated cathodic protection properties of Ag/NiS/TiO_2_ nanocomposites

**DOI:** 10.1038/s41598-022-08929-z

**Published:** 2022-03-21

**Authors:** Ning Wang, Jing Wang, Yanli Ning, Chengyue Ge, Baorong Hou, Qianyu Zhao, Yiteng Hu

**Affiliations:** 1 CAS Key Laboratory of Marine Environmental Corrosion and Bio-Fouling, Institute of Oceanology, Chinese Academy of Sciences, No.7 Nanhai Road, Qingdao, 266071 People’s Republic of China; 2 Open Studio for Marine Corrosion and Protection, Pilot National Laboratory for Marine Science and Technology, Qingdao, 266237 People’s Republic of China; 3 Center for Ocean Mega-Science, Chinese Academy of Sciences, No. 7 Nanhai Road, Qingdao, 266071 People’s Republic of China

**Keywords:** Nanoscale materials, Electronic properties and materials, Nanoparticles, Nanowires

## Abstract

TiO_2_ is a semiconductor material used in photoelectric conversion. In order to improve its light utilization rate, nickel sulfide and silver nanoparticles were synthesized on the surface of titanium dioxide nanowires by simple impregnation-deposition and photoreduction methods. A series of studies were conducted on the cathodic protection effect of Ag/NiS/TiO_2_ nanocomposites on 304 stainless steel, with additional analyses on the material’s morphology, composition, and light absorption characteristics. The results indicate that when the number of nickel sulfide impregnation-deposition cycles is 6, and the silver nitrate photoreduction concentration is 0.1 M, the prepared Ag/NiS/TiO_2_ nanocomposites can provide the best cathodic protection for 304 stainless steel.

## Introduction

The application of n-type semiconductors to photocathodic protection using sunlight has been a hot topic in recent years. Under the excitation of sunlight, the electrons in the valence band (VB) of the semiconductor material will be excited to the conduction band (CB), generating photogenerated electrons. If the conduction band potential of the semiconductor or nanocomposite is more negative than the self-etching potential of the coupled metal, these photogenerated electrons will be transferred to the surface of the coupled metal. The accumulation of electrons will lead to cathode polarization of the metal, and cathodic protection of the coupled metal will be provided^1–7^. Semiconductor materials are theoretically regarded as a nonsacrificial photoanode because the anodic reaction does not decompose the semiconductor materials themselves while is carried out through water oxidation by photogenerated holes or adsorbed organic pollutants^1^, or the presence of trapping agents captures photogenerated holes. Most importantly, the semiconductor material should have a CB potential that is more negative than the corrosion potential of the protected metal. Only in this way can photogenerated electrons be transferred from the semiconductor's conduction band to the protected metal. Photochemical corrosion resistance studies have focused on inorganic n-type semiconductor materials with wide band gaps (3.0–3.2EV)^1–7^, which are only responsive to ultraviolet light (< 400 nm), reducing the availability of light.

In the field of marine anticorrosion, photo-electrochemical cathodic protection technology plays a pivotal role. TiO_2_ is a kind of semiconductor material with excellent UV-light-absorbing property and photocatalysis performance. However, due to its low light utilization rate, photogenerated electron holes easily compound and cannot be protected under dark-state conditions. Further study is needed to propose reasonable and practical solutions. It has been reported that many surface modification treatments can be applied to improve the photosensitivity of TiO_2_, such as doping with Fe, N and compositing with Ni_3_S_2_, Bi_2_Se_3_, CdTe and so on. Therefor, compositing TiO_2_ with high photoelectric conversion efficiency materials has been widely applied in the field of photogenerated cathodic protection.

Nickel sulfide is a semiconductor material with a narrow band gap width of only 1.24 eV^8,9^. The narrower the band gap width is, the stronger the utilization ratio of light will be. When nickel sulfide is compounded onto the surface of titanium dioxide, the utilization ratio of light can be widened. Combined with titanium dioxide, the separation efficiency of photogenerated electrons and holes can be improved effectively. Nickel sulfide is widely used in electrocatalytic hydrogen production, batteries, and pollutant degradation^8–10^. However, its application in photocathodic protection has not been reported. In this study, a semiconductor material with a narrow band gap width was chosen for solving the low light utilization efficiency of TiO_2_. Nickel sulfide and silver nanoparticles were combined on the surface of TiO_2_ nanowires by impregnation-deposition and photoreduction methods, respectively. The Ag/NiS/TiO_2_ nanocomposite improves the efficiency of light utilization, which makes the absorption range of light expand from the ultraviolet region to the visible region. Meanwhile, the deposition of silver nanoparticles gives the Ag/NiS/TiO_2_ nanocomposites excellent optical stability and sustainable cathodic protection.

## Experimental

### Preparation of Ag/NiS/TiO_2_ nanocomposite

#### Preparation of titanium dioxide nanowires

First, a 0.1 mm thick titanium foil with a purity of 99.9% was cut to a size of 30 mm × 10 mm for the experiment. Then, the titanium foil was polished with 2500-mesh sandpaper 100 times on each surface and then washed with acetone, anhydrous ethanol, and distilled water in turn. The titanium plates were placed in a mixture at 85 °C (sodium hydroxide:sodium carbonate:water = 5:2:100) for 90 min, removed, and cleaned with distilled water. The surface was etched by a HF solution (HF:H_2_O = 1:5) for 1 min, and then cleaned with acetone, ethanol, and distilled water in turn, finally blow-dried for later use. Titanium dioxide nanowires were rapidly prepared on the surface of titanium foil by one-step anodic oxidation^11^. A traditional two-electrode system was used for anodic oxidation, the working electrode was a titanium sheet, and the counter electrode was a platinum electrode. The titanium plates were placed into 400 mL of a 2 M sodium hydroxide solution with an electrode clamp. The current of the DC power supply was stable at approximately 1.3 A. The solution temperature was kept at 80 °C for 180 min during the reaction of the system. The titanium sheet was removed, cleaned with acetone and ethanol, cleaned with distilled water, and dried naturally. Then, the sample was placed in a muffle furnace at 450 °C (heating rate of 5 °C/min), kept at a constant temperature for 120 min, and placed in a dry dish.

#### Preparation of NiS/TiO_2_ nanocomposite

The composite of nickel sulfide and titanium dioxide was obtained by a simple and easy impregnation-deposition method^8^. First, nickel nitrate (0.03 M) was dissolved in ethanol under magnetic stirring for 20 min to produce an ethanol solution of nickel nitrate. Next, sodium sulfide (0.03 M) was prepared with a mixed methanol solution (methanol:water = 1:1). Then titanium dioxide tablets were put into the above-prepared solution, removed after 4 min, and rinsed quickly with the mixed solution of methanol and water (methanol:water = 1:1) for 1 min. After the surface was dried, the tablet was placed in the muffle furnace under vacuum at 380 °C for 20 min, cooled to room temperature and dried. The number of cycles was 2, 4, 6, and 8.

#### Preparation of Ag/NiS/TiO_2_ nanocomposite

**Figure 1:**
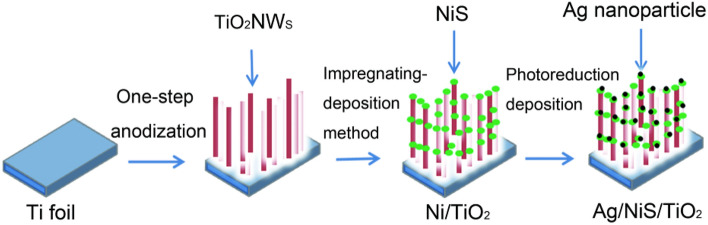
Schematic diagram of the procedures used to prepare the Ag/NiS/TiO_2_ nanocomposite.

Silver nanoparticles modified Ag/NiS/TiO_2_ nanocomposite by photoreduction^12,13^. The obtained Ag/NiS/TiO_2_ nanocomposite was put into the silver nitrate solution required for the experiment. Then, the sample was exposed to ultraviolet light for 30 min, its surface was washed clean with deionized water, and the Ag/NiS/TiO_2_ nanocomposite was obtained by natural drying. The above experimental process is shown as Fig. 1.

### Characterization of Ag/NiS/TiO_2_ nanocomposite

The Ag/NiS/TiO_2_ nanocomposite was mainly characterized by field-emission scanning electron microscopy (FESEM), energy dispersive spectroscopy (EDS), X-ray photoelectron spectroscopy (XPS), and UV–visible diffuse reflectance (UV–Vis). FESEM was performed using a Nova NanoSEM 450 microscope (FEI Company, USA). Its acceleration voltage is 1 kV, and spot size is 2.0. The equipment uses a CBS probe to receive secondary electrons and backscattered electrons to analyze the morphology. EDS was performed with an Oxford X-Max N50 EDS system (Oxford Instruments Technology Co., Ltd.) with an acceleration voltage of 15 kV and a spot size of 3.0. Qualitative and quantitative analyses were conducted with characteristic X-ray. X-ray photoelectron spectroscopy was performed using an Escalab 250Xi spectrometer (Thermo Fisher Scientific Corporation, USA) operated in fixed energy mode with an excitation power of 150 W, monochromatic Al Kα radiation (1486.6 eV) were used as the excitation source. Full scanning range of 0–1600 eV, general energy of 50 eV, step width of 1.0 eV, and contaminated carbon (~ 284.8 eV) were used as the binding energy charge correction reference. The pass energy of the narrow scan was 20 eV, and the step width was 0.05 eV. UV–visible diffuse reflectance spectroscopy was performed with a Cary 5000 spectrometer (Varian, USA) with a barium sulfate reference plate and a scanning range of 10°–80°.

### Photoelectric properties test of Ag/NiS/TiO_2_ nanocomposites

#### Preparation of 304 stainless steel working electrode

In this work, the composition (wt.%) of 304 stainless steel was 0.08 C, 1.86 Mn, 0.72 Si, 0.035 P, 0.029 s, 18.25 Cr, 8.5 Ni, and with Fe forming the remainder. The 304 stainless steel with dimensions of 10 mm × 10 mm × 10 mm was sealed with epoxy resin, leaving the exposed surface area 1 cm^2^. Its surface was polished with 2400 mesh silicon carbide sandpaper and washed with ethanol. Afterward, the stainless steel was ultrasonicated in deionized water for 5 min, then stored in a drying dish.

#### Open circuit potential (OCP) test

**Figure 2:**
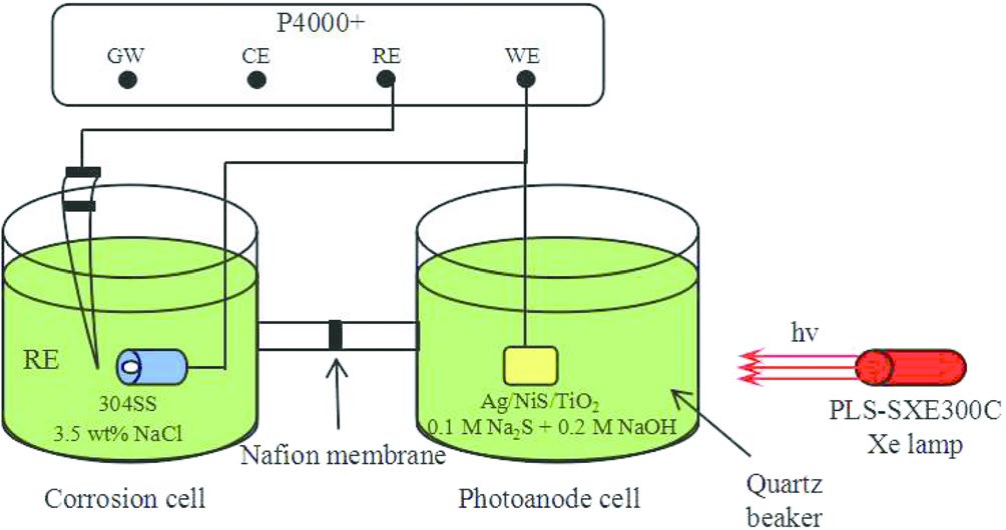
Schematic illustration of the experimental set-up for measuring photoinduced potential.

During the OCP experiment, the 304 stainless steel and Ag/NiS/TiO_2_ photoanode were placed in the corrosion pool and photoanode cell, respectively (Fig. 2). The corrosion cell was filled with 3.5% NaCl solution whereas the 0.25 M Na_2_SO_3_ was filled into the photoanode cell as a hole trapping agent. A naphthol membrane was used to separate the two electrolytes from mixing. OCP was measured by an electrochemical workstation (P4000+, USA). The reference electrode was a saturated calomel electrode (SCE). The light source (xenon lamp, PLS-SXE300C, Poisson Technologies Co., Ltd.) and 420 cut-off plates were placed at the outlet of the light source so that visible light reached the optical anode through quartz glass. The 304 stainless steel electrode was connected with the photoanode by a copper wire. Before the experiment, the 304 stainless steel electrode was immersed in a 3.5% NaCl solution for 2 h to ensure that the steady state was achieved. At the beginning of the experiment, by opening and closing the light, the excited electrons of the photoanode will reach the surface of 304 stainless steel through the wire. After switching on the light, the OCP of 304 stainless steel was recorded.

#### Photocurrent density test

**Figure 3:**
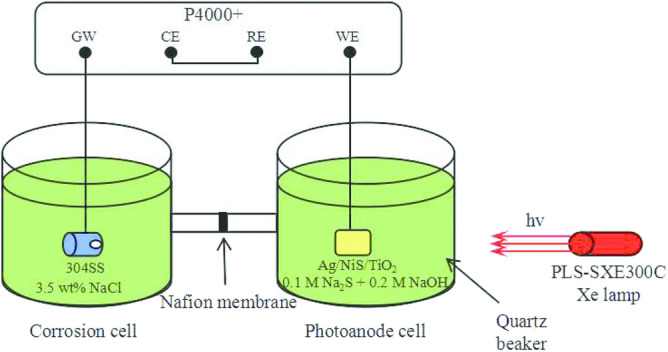
Schematic illustrations of the experimental set-up for measuring photoinduced current density.

During the photoinduced current density experiment, 304SS and Ag/NiS/TiO_2_ photoanode were placed in the corrosion cell and photoanode cell, respectively (Fig. 3). The photocurrent density was measured with the same apparatus as OCP. To obtain the actual photocurrent density between 304 stainless steel and photoanode, the potentiostat was adopted as a zero-resistance ammeter that connected between 304 stainless steel and photoanode under a non-polarization condition. In order to achieve this goal, the reference electrode and the counter electrode are short-connected in the experimental device, so that the electrochemical workstation is like a zero-resistance ammeter, and the true current density can be measured. The 304 stainless steel electrode is connected to the grounding position of the electrochemical workstation, and the photoanode is connected to the working electrode clip. At the beginning of the experiment, by opening and closing the light, the excited electrons of the photoanode will reach the surface of 304 stainless steel through the wire. At this time, the change of the photocurrent density on the surface of 304 stainless steel can be observed.

## Results and discussion

### Cathodic protection performance of Ag/NiS/TiO_2_ nanocomposite

In order to investigate the cathodic protection performance of nanocomposite on 304 stainless steel, the change in the photoionization potential of 304 stainless steel coupled with nanocomposite and the change in photoionization current density between the nanocomposite and 304 stainless steel were tested.

**Figure 4:**
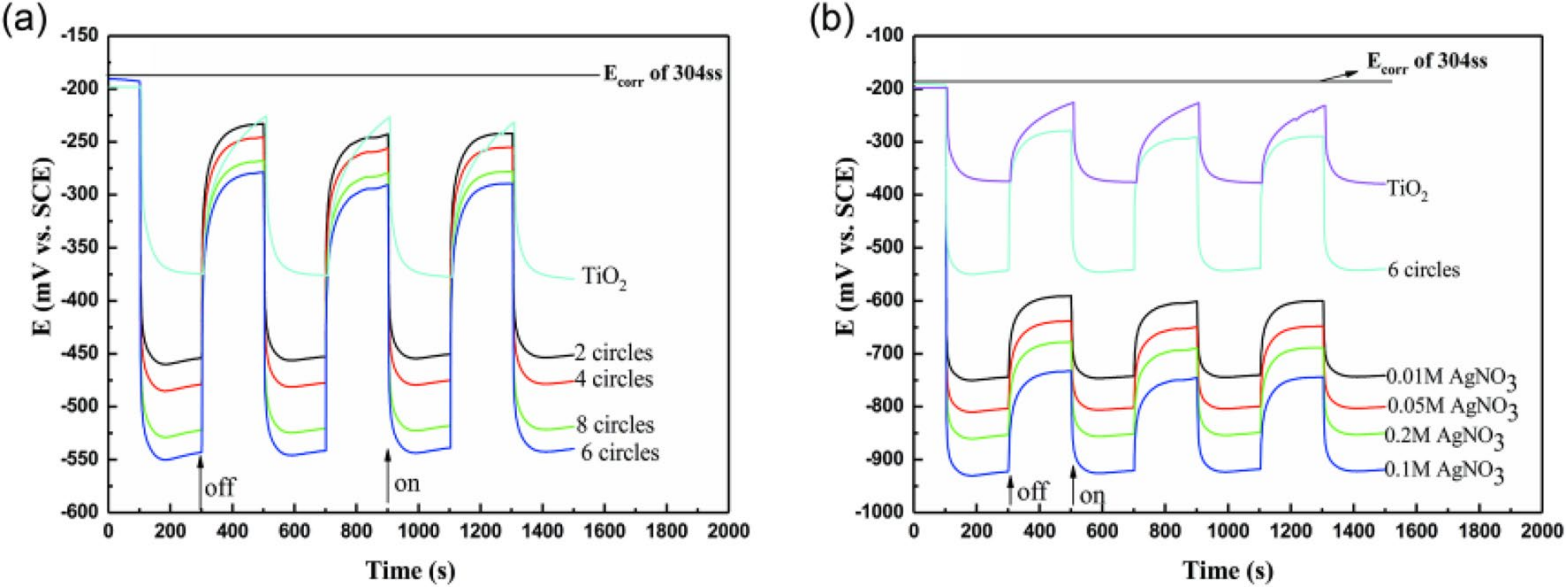
OCP variations of the 304 SS electrode coupled with the NiS/TiO_2_ nanocomposite (**a**) and Ag/NiS/TiO_2_ nanocomposite (**b**) with and without illumination (λ > 400 nm).

Figure 4 shows the open-circuit potential variation of 304 stainless steel coupled with nanocomposites under visible light irradiation and dark state conditions. Figure 4a shows the influence of nickel sulfide impregnation-deposition times on the open-circuit potential, while Fig. 4b shows the influence of the silver nitrate concentration on the open-circuit potential during photoreduction. Figure 4a shows that compared to the nickel sulfide composite, the open-circuit potential of NiS/TiO_2_ nanocomposite coupled with 304 stainless steel is significantly reduced at the moment that the lamp is opened. In addition, the open-circuit potential is more negative than that of pure titanium dioxide nanowire, indicating that the nickel sulfide composite produces more electrons and improves the photocathodic protection effect of titanium dioxide. However, when light exposure ends, the open-circuit potential rises rapidly to the open-circuit potential of stainless steel, indicating that nickel sulfide does not have an energy storage effect. The influence of the number of impregnation-deposition cycles on the open-circuit potential can be observed in Fig. 4a. When the deposition time is 6, the extreme electrical potential of the nanocomposite reaches − 550 mV relative to the saturated calomel electrode, and the potential of the nanocomposite deposited 6 times is significantly more negative than that of the nanocomposite under other conditions. Thus, the NiS/TiO_2_ nanocomposite obtained after 6 deposition cycles provides the best cathodic protection for 304 stainless steel.

As shown in Fig. 4b, when light is applied, the open-circuit potential of 304 stainless steel coupled with Ag/NiS/TiO_2_ nanocomposite was significantly reduced. After surface deposition of silver nanoparticles, the open-circuit potential was significantly reduced compared with pure TiO_2_ nanowires. The NiS/TiO_2_ nanocomposite potential was more negative, showing that the cathodic protection effect of titanium dioxide improves significantly after the deposition of silver nanoparticles. The open-circuit potential increases rapidly when light exposure ends, compared with the saturated calomel electrode, the open-circuit potential can reach − 580 mV, which is lower than 304 stainless steel’s potential (− 180 mV). This result shows that the nanocomposites have a noticeable energy storage effect after the deposition of silver particles on their surfaces. Figure 4b also shows the influence of the silver nitrate concentration on the open-circuit potential. When the concentration of silver nitrate is 0.1 M, the electric extreme potential relative to the saturated calomel electrode reaches − 925 mV. After 4 application cycles, the potential remained at the level after the first application, indicating that the nanocomposite has excellent stability. Thus, when the concentration of silver nitrate was 0.1 M, the Ag/NiS/TiO_2_ nanocomposite obtained provided the best cathodic protection for 304 stainless steel.

With the increase in the deposition times of nickel sulfide, the deposition of nickel sulfide on the surface of titanium dioxide nanowires gradually improved. When visible light irradiated the surface of the nanowires, more nickel sulfide active sites were excited to generate electrons, and the photoionization potential decreased more. However, when too many nickel sulfide nanoparticles are deposited on the surface, the excited nickel sulfide decreases instead, which is not conducive to light absorption^3^. After the silver particles are deposited on the surface, due to the surface plasmon resonance effect of the silver particles, the generated electrons will quickly transfer to the surface of 304 stainless steel, producing an excellent cathodic protection effect. When too many silver particles are deposited on the surface, the silver particles will become the composite point of photoelectrons and holes, which is adverse to the production of photoelectrons^14^. In summary, the Ag/NiS/TiO_2_ nanocomposite can provide the best cathodic protection for 304 stainless steel after 6 nickel sulfide depositions at 0.1 M of silver nitrate.

**Figure 5:**
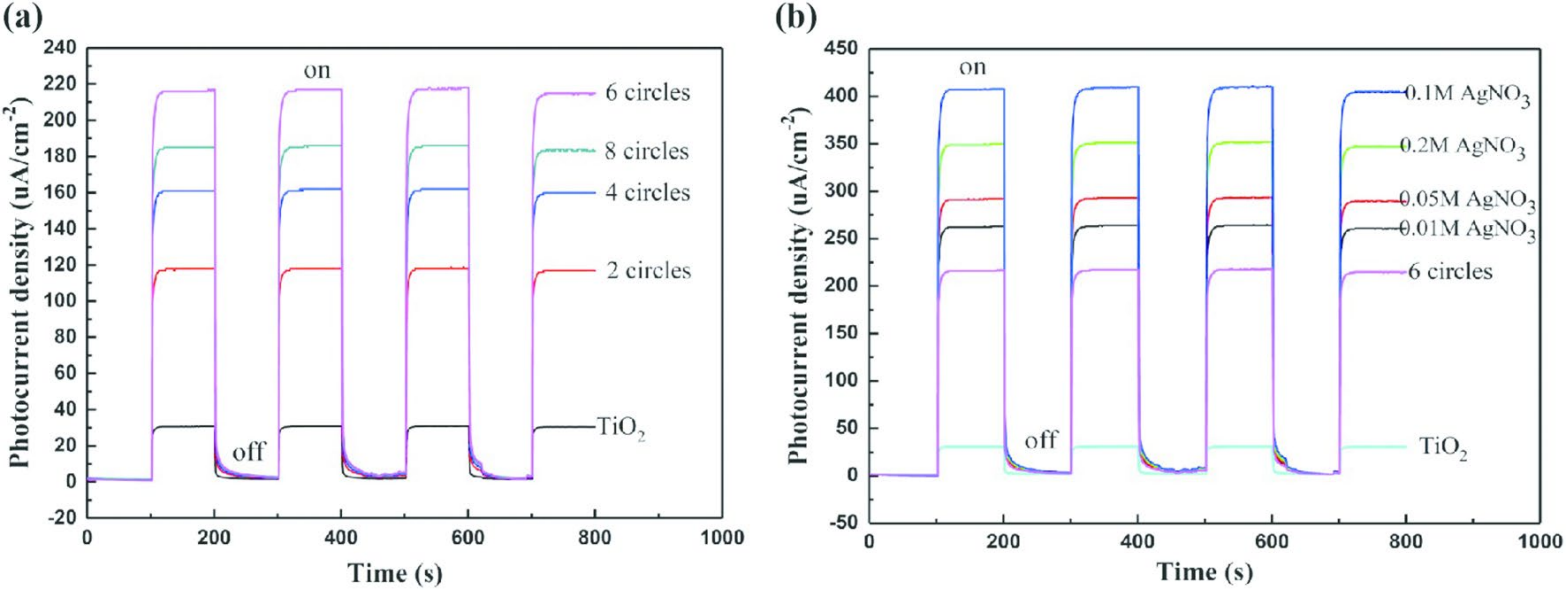
Photocurrent density variations of the 304 SS electrode coupled with the NiS/TiO_2_ nanocomposite (**a**) and Ag/NiS/TiO_2_ nanocomposite (**b**) with and without illumination (λ > 400 nm).

The magnitude of the photocurrent density represents the separation ability of photogenerated electrons and holes, and a more significant photocurrent density represents a stronger separation ability of photogenerated electrons and holes. There are many studies showing that NiS has been widely used in synthetic photocatalytic materials to improve the ability of material optoelectronics and hole separation^15–20^. The noble-metal-free graphene and NiS co-modified g-C_3_N_4_ composite was studied by Chen et al.^15^. The maximum photocurrent intensity of the modified g-C_3_N_4_/0.25%RGO/3%NiS was 0.018 µA/cm^2^. The photocurrent density of CdSe-NiS with a value of about 10 µA/cm^2^ was studied by Chen et al.^16^. The CdS@NiS composites with a 15 µA/cm^2^ photocurrent density was synthesized by Liu et al.^18^. However, NiS applied in photocathodic protection has not been reported. In our study, the photocurrent density of TiO_2_ is significantly improved by NiS modification. Figure 5 shows the variation in the photocurrent densities of 304 stainless steel and nanocomposite under visible and no illumination conditions. As shown in Fig. 5a, the photocurrent density of the NiS/TiO_2_ nanocomposite increases rapidly at the moment when the light is switched on, and the photocurrent density is positive, indicating that electrons flow from the nanocomposite through the electrochemical workstation to the surface of 304 stainless steel. After the nickel sulfide composite is prepared, the photocurrent density is more substantial than that of pure titanium dioxide nanowires. The photocurrent density of nickel sulfide reached 220 μA/cm^2^, 6.8 times higher than that of titanium dioxide nanowires (32 μA/cm^2^) when the number of nickel sulfide immersion and deposition was 6. As shown in Fig. 5b, upon turning on the xenon lamp, the photocurrent density between the Ag/NiS/TiO_2_ nanocomposite and 304 stainless steel was significantly higher than that between pure titanium dioxide and the NiS/TiO_2_ nanocomposite. Figure 5b also shows the influence of the concentration of silver nitrate on the photocurrent density during the photoreduction process. When the concentration of silver nitrate is 0.1 M, its photocurrent density reaches 410 μA/cm^2^, which is 12.8 times the photocurrent density generated by titanium dioxide nanowires (32 μA/cm^2^) and 1.8 times that of the NiS/TiO_2_ nanocomposite. A heterogeneous junction electric field forms at the Ag/NiS/TiO_2_ nanocomposite interface, making it easier to separate photogenerated electrons from holes.

In summary, through 6 nickel sulfide maceration-deposition cycles under 0.1 M concentrated silver nitrate, the photocurrent density between the Ag/NiS/TiO_2_ nanocomposite and 304 stainless steel reached 410 μA/cm^2^, and the extreme electrical potential relative to the saturated calomel electrode reached − 925 mV. Under such conditions, 304 stainless steel coupled with Ag/NiS/TiO_2_ could provide the best cathodic protection.

### Surface morphology analysis of Ag/NiS/TiO_2_ nanocomposite

**Figure 6:**
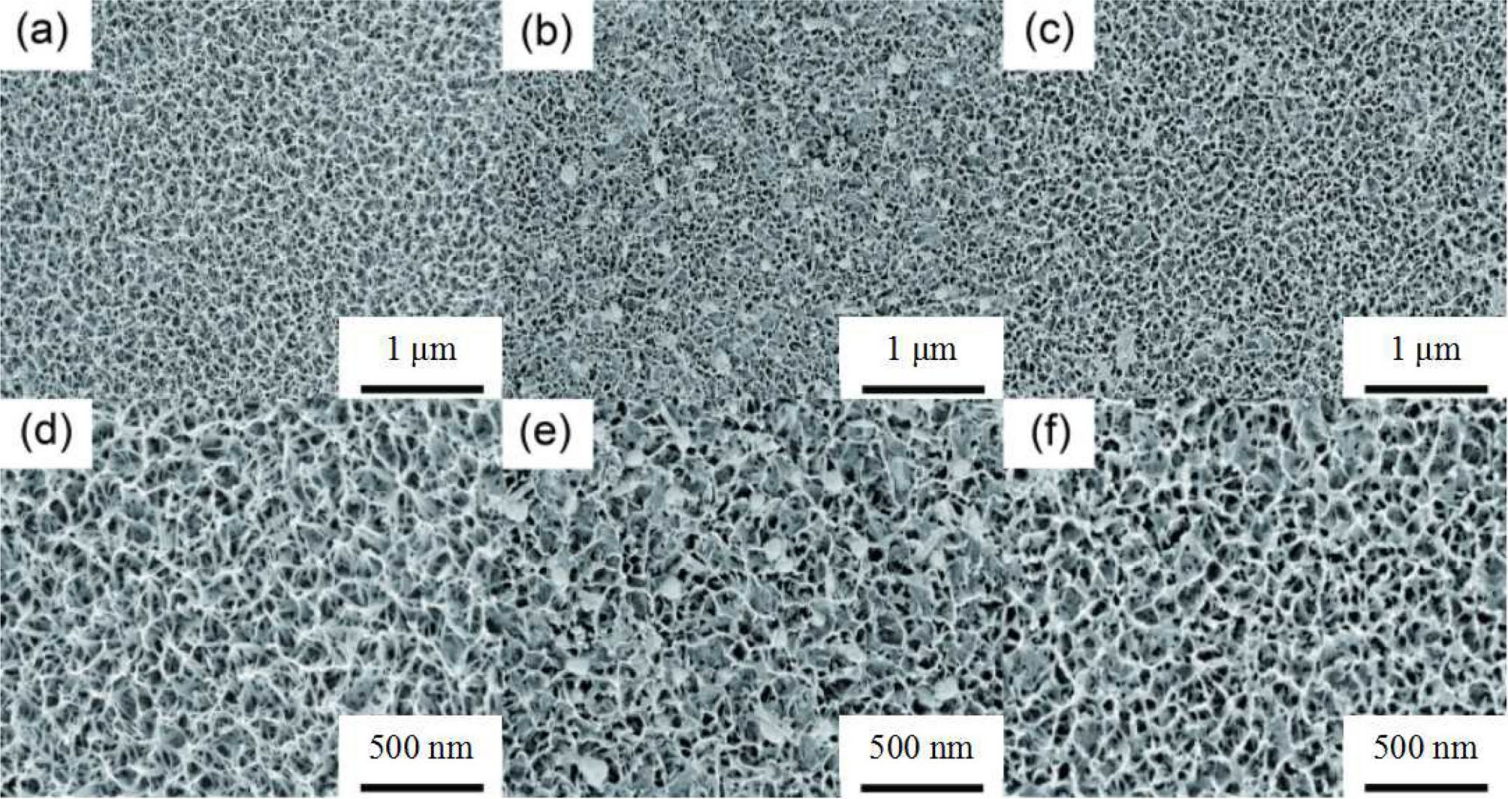
SEM images of pure TiO_2_ (**a**,**d**), NiS/TiO_2_ nanocomposite with 6 NiS impregnation-deposition cycles (**b**,**e**) and Ag/NiS/TiO_2_ nanocomposite with 6 NiS impregnation-deposition cycles performed at a AgNO_3_ concentration of 0.1 M (**c**,**f**).

**Figure 7:**
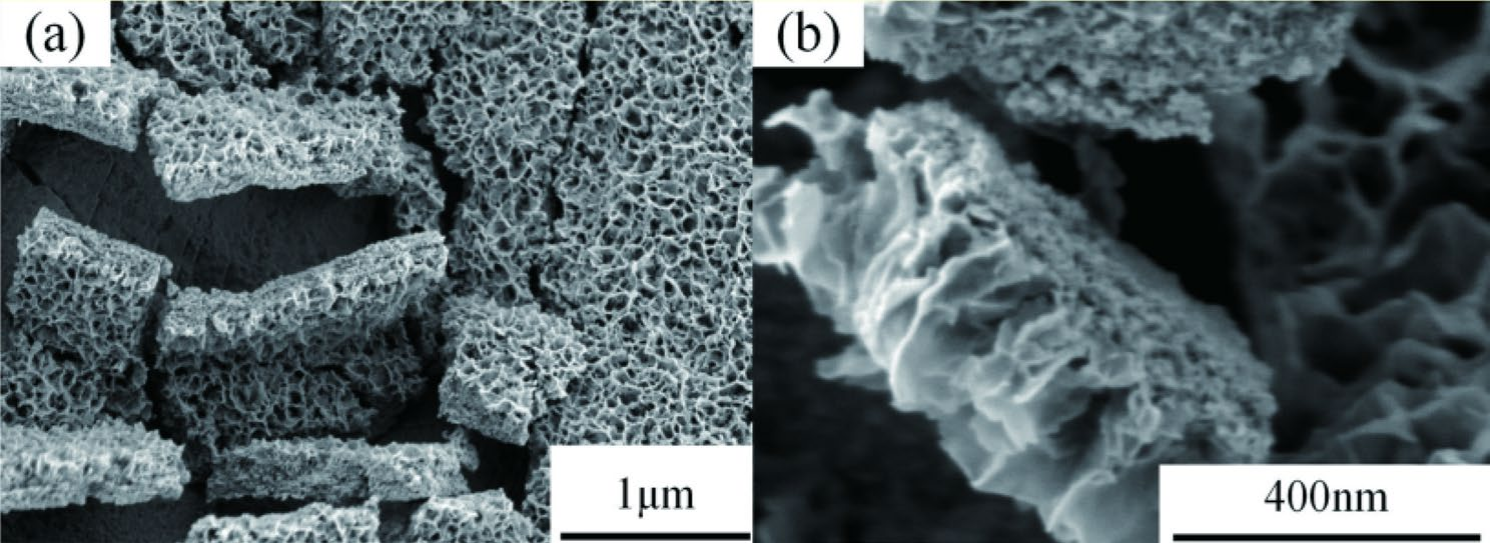
Cross-section of Ag/NiS/TiO_2_ nanofilms with 6 NiS impregnation-deposition cycles performed at a AgNO_3_ concentration of 0.1 M.

Figure 6 shows the surface electron microscopy images of pure titanium dioxide nanowire, nickel sulfide composite nanoparticle, and silver nanoparticle under optimal conditions. Figure 6a,d show the pure titanium dioxide nanowire prepared by one-step anodic oxidation. The surface distribution of titanium dioxide nanowire is uniform, the nanowire structures are close to each other, and the pore size distribution is uniform. Figure 6b,e are titania electron micrographs of nickel sulfide composites impregnated-deposited 6 times. From Fig. 6e of electron microscopy images taken at 200,000 times magnification, we can see that the nickel sulfide composite nanoparticles are relatively uniform, and their particle size is relatively large, their diameter is approximately 100–120 nm. Some nanoparticles can be observed in the nanowire space position, and titanium dioxide nanowires are clearly visible. Figure 6c,f show the electron microscopy images of the NiS/TiO_2_ nanocomposite when the concentration of silver nitrate was 0.1 M. Comparing with Fig. 6b and Fig. 6e, Fig. 6c and Fig. 6f show that silver nanoparticles are deposited on the surface of the composite, the distribution of silver nanoparticles is uniform, and that their diameter is approximately 10 nm. Figure 7 shows the cross-section of Ag/NiS/TiO_2_ nanofilms with 6 NiS impregnation-deposition cycles performed at a AgNO_3_ concentration of 0.1 M. From the higher-magnification image, the film thickness is measured to be 240–270 nm. In summary, nickel sulfide and silver nanoparticles composite on the surface of titanium dioxide nanowires.

**Figure 8:**
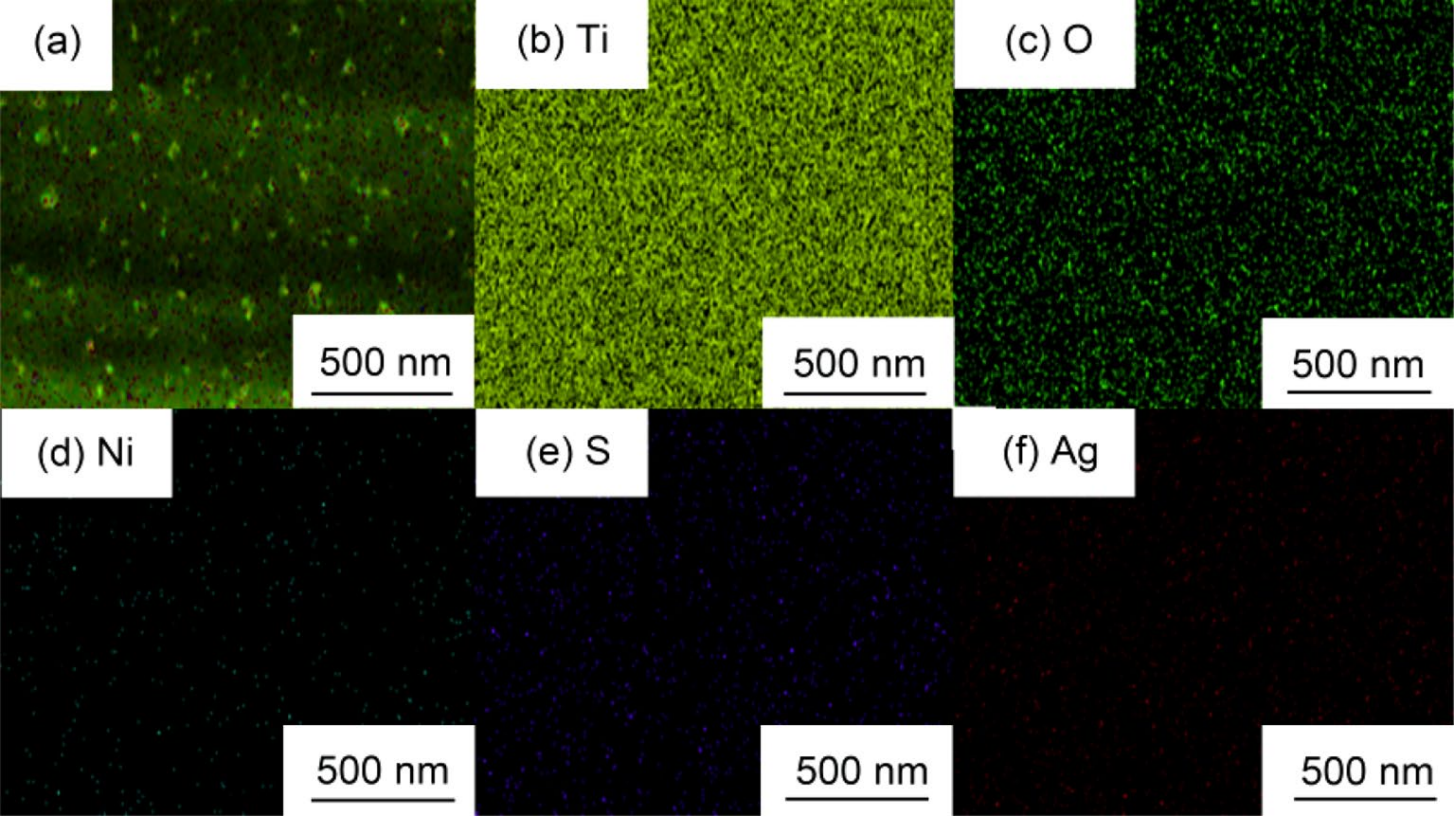
Elemental distributions (Ti, O, Ni, S and Ag) of the Ag/NiS/TiO_2_ nanocomposite with 6 NiS impregnation-deposition cycles performed at a AgNO_3_ concentration of 0.1 M.

Figure 8 shows the elemental surface distribution of the Ag/NiS/TiO_2_ nanocomposite obtained by performing 6 nickel sulfide impregnation-deposition cycles at a silver nitrate concentration of 0.1 M. The elemental surface distribution shows that the energy spectrum detects Ti, O, Ni, S, and Ag. In terms of content, Ti and O are the most abundant elements in the distribution, while the Ni and S contents are approximately the same, but their contents are significantly less than the Ag content. It can also be proven that the number of composite silver nanoparticles on the surface is greater than that of nickel sulfide. The uniform distribution of each element on the surface indicates that nickel sulfide and silver are uniformly combined on the surface of titanium dioxide nanowires. To analyse the specific composition and the combination state of the substance, we further performed X-ray photoelectron spectroscopy.

### Surface state analysis of Ag/NiS/TiO_2_ nanocomposite

**Figure 9:**
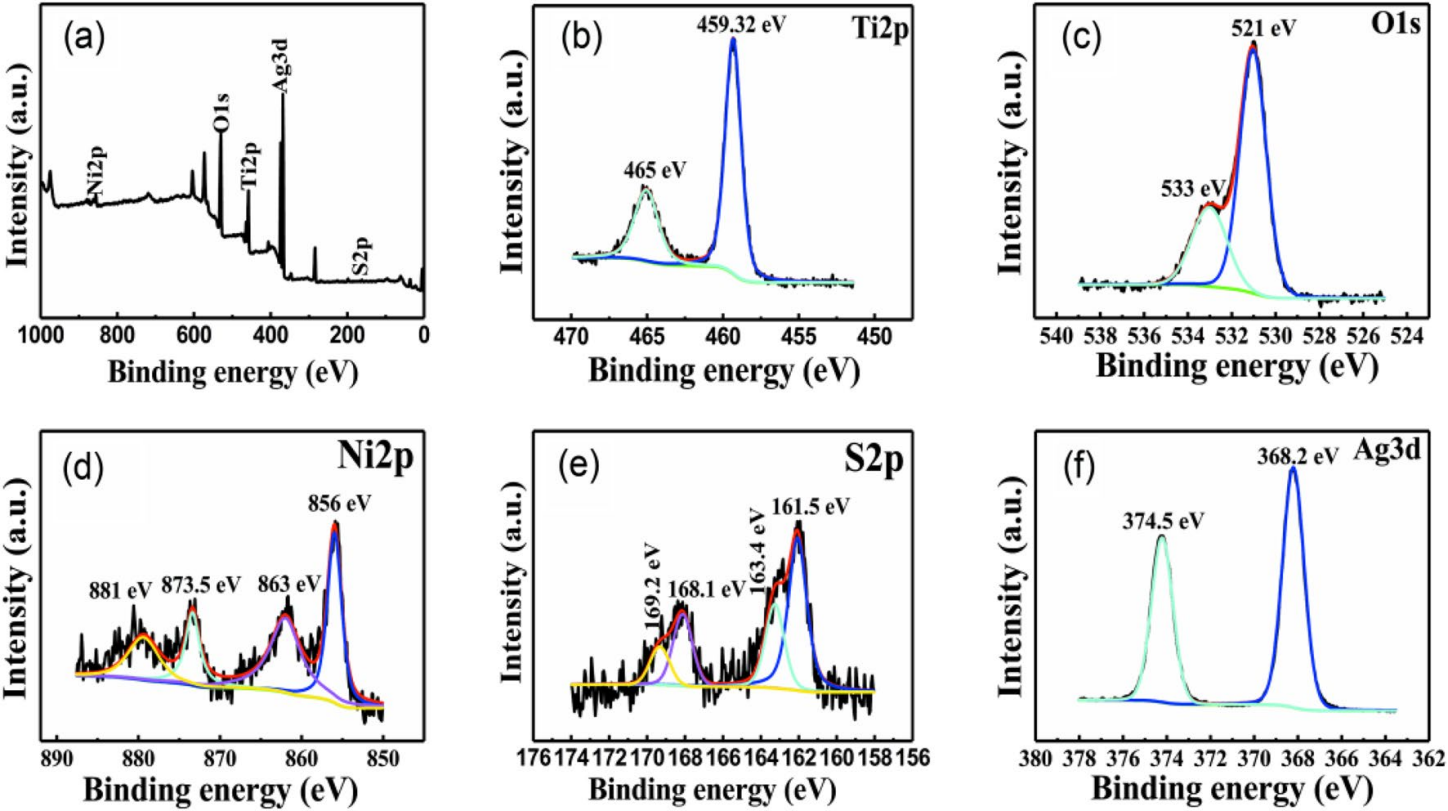
XPS survey spectra of Ag/NiS/TiO_2_ (**a**) and high-resolution Ti 2p (**b**), O 1 s (**c**), Ni 2p (**d**), S 2p (**e**) and Ag 3d (**f**) XPS spectra.

Figure 9 shows the XPS spectra of the Ag/NiS/TiO_2_ nanocomposite prepared by performing 6 nickel sulfide impregnation-deposition cycles at 0.1 M of silver nitrate., in which Fig. 9a is the full spectrum, and the other spectra are the high-resolution spectra of the elements. As seen from the full spectrum of Fig. 9a, absorption peaks of Ti, O, Ni, S and Ag were detected in the nanocomposite material, and the existence of these five elements was proven. The detection results were consistent with those of EDS. The redundant peaks in Fig. 9a are the carbon peaks used to correct the binding energy of the sample. Figure 9b shows the high-resolution Ti energy spectrum. The absorption peaks of the 2p orbital are located at 459.32 and 465 eV, and the absorption peaks at these two places correspond to the absorption of Ti 2p_3/2_ and Ti 2p_1/2_ orbitals^1^. The two absorption peaks prove that the valence of titanium is Ti^4+^, corresponding to Ti in TiO_2_.

Figure 9d shows the high-resolution Ni energy spectrum, and there are four absorption peaks for the Ni 2p orbital. The absorption peaks at 856 and 873.5 eV correspond to Ni 2p_3/2_ and Ni 2p_1/2_ orbitals^8,10^, where the absorption peaks are derived from NiS. The absorption peaks at 881 and 863 eV were derived from nickel nitrate and were caused by the nickel nitrate reagent during sample preparation. Figure 9e shows the high-resolution S energy spectra. The absorption peaks of the S 2p orbital are located at 161.5 and 168.1 eV, corresponding to S 2p_3/2_ and S 2p_1/2_ orbitals^21–24^. These two peaks are attributed to nickel sulfide compounds. The absorption peaks at 169.2 and 163.4 eV were derived from the sodium sulfide reagent. Figure 9f shows the high-resolution Ag energy spectrum, in which the 3d orbital absorption peaks of silver are located at 368.2 and 374.5 eV, and the absorption peaks at the two places correspond to the absorption of Ag 3d_5/2_ and Ag 3d_3/2_ orbitals^12,13^. The peaks at these two places prove that silver nanoparticle exist in the elemental silver state. In summary, the nanocomposites are mainly composed of Ag, NiS, and TiO_2_, as determined by X-ray photoelectron spectroscopy, proving that nickel sulfide and silver nanoparticles are successfully composited on the surface of titanium dioxide nanowires.

### Optical absorbability analysis of Ag/NiS/TiO_2_ nanocomposites

**Figure 10:**
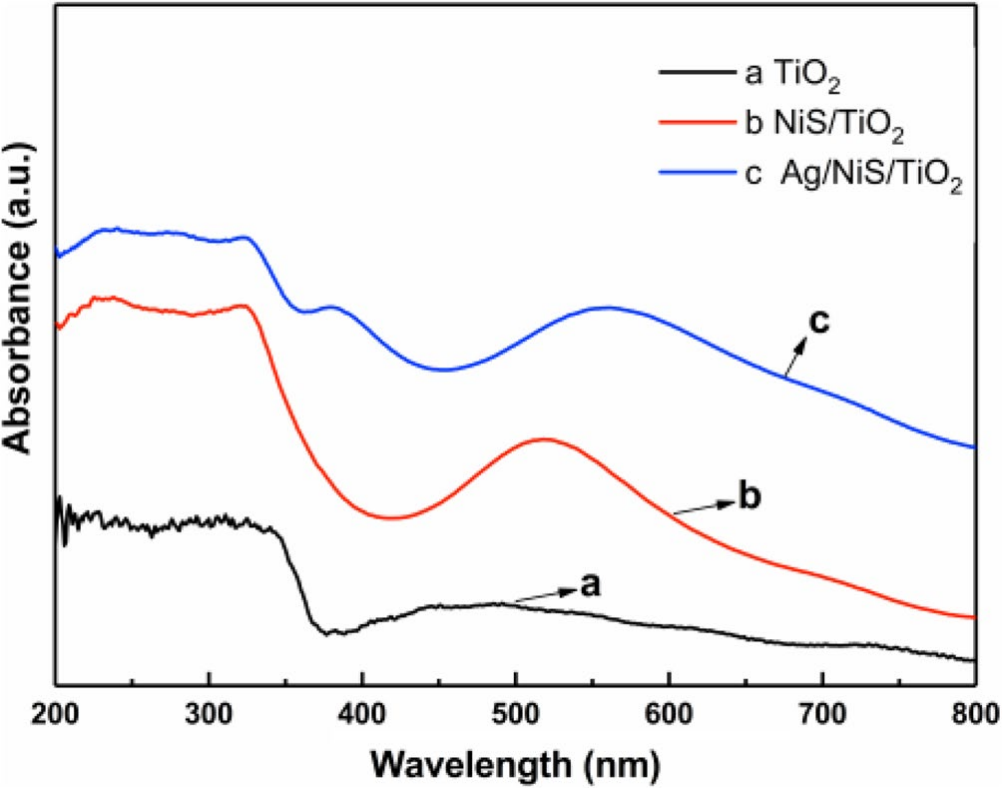
UV/Vis diffuse reflectance spectra of the prepared TiO_2_ NWs, NiS/TiO_2_ nanocomposite and Ag/NiS/TiO_2_ nanocomposite.

Figure 10 shows the UV–Vis diffuse reflectance spectra of the as-prepared titanium dioxide nanowires, NiS/TiO_2_ nanocomposites, and Ag/NiS/TiO_2_ nanocomposites. As seen from the figure, the absorption threshold of titanium dioxide nanowires is approximately 390 nm, and the absorbed light is mainly concentrated on the ultraviolet region. The figure shows that the light adsorbed extends to the visible region after nickel sulfide and silver nanoparticles are composite on the surface of^21,22^ titanium dioxide nanowires. At the same time, the absorption of ultraviolet light by nanocomposites is enhanced, which is due to the narrow band gap of nickel sulfide. The narrower the band gap is, the lower the energy barrier of the electron transition and the higher the utilization of light. After silver nanoparticles were composited on the NiS/TiO_2_ surface, the absorption intensity and wavelength of light did not increase significantly, mainly due to the plasma resonance effect on the surface of silver nanoparticles. The absorption wavelength of titanium dioxide nanowires could not be significantly improved compared to nickel sulfide composite nanoparticles with narrow band gap widths. In summary, after nickel sulfide and silver nanoparticles were composited on the surface of titanium dioxide nanowires, their optical absorption properties were significantly enhanced, and the absorption range of light was extended from the ultraviolet region to the visible region, which increased the utilization rate of light and improved the photoelectron generation ability of the material.

### Mechanistic analysis of Ag/NiS/TiO_2_ nanocomposite

**Figure 11:**
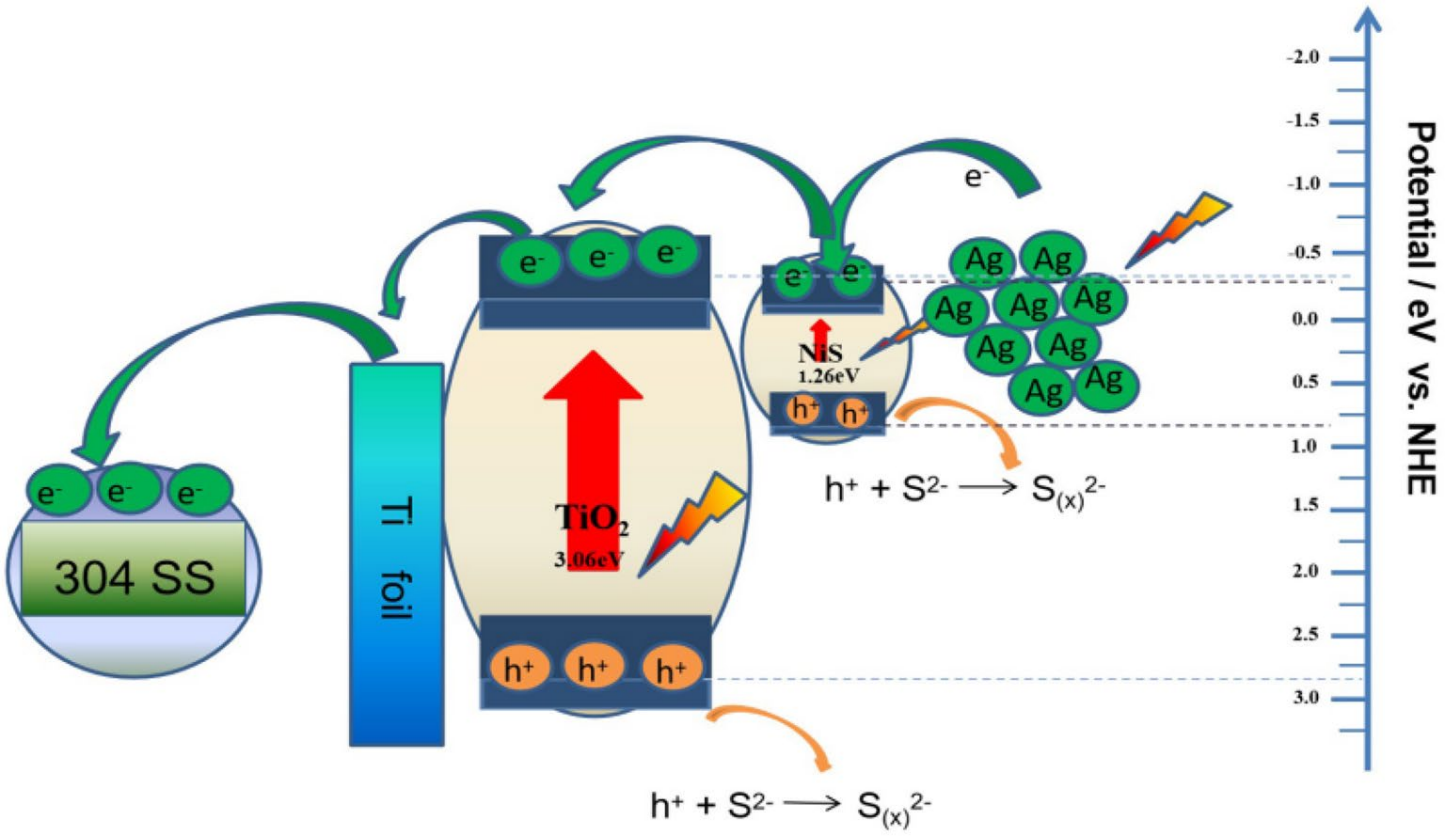
Schematic diagram of the photoelectrochemical anticorrosion process in the Ag/NiS/TiO_2_ nanocomposites under visible light.

Figure 11 shows the photochemical corrosion resistance mechanism of the Ag/NiS/TiO_2_ nanocomposite under visible light irradiation. A feasible diagram of the corrosion resistance mechanism is proposed according to the potential distribution^10,21,24^ of the silver, nickel sulfide nanoparticle, and the titanium dioxide conduction band. Since the conduction band potential of silver nanoparticles is negative with respect to that of nickel sulfide and the conduction band potential of nickel sulfide is negative compared to that of titanium dioxide, the electron flow direction is approximately Ag → NiS → TiO_2_ → 304 stainless steel. When light is irradiated on the surface of the nanocomposite materials, silver nanoparticles can rapidly produce photogenerated holes and electrons due to the surface plasmonic resonance effect of silver nanoparticles, and photogenerated electrons rapidly transition from the valence band to the conduction band position due to the excitation of titanium dioxide and nickel sulfide. Since the conductivity of silver nanoparticles is more negative than that of nickel sulfide, the electrons in the CB of silver nanoparticles rapidly transition to the CB of nickel sulfide. The conductance potential of nickel sulfide is more negative than that of titanium dioxide, so the electrons of nickel sulfide and silver conductance quickly accumulate in the CB of titanium dioxide. The generated photogenerated electrons reach the surface of 304 stainless steel through the titanium matrix, and the enriched electrons participate in the cathode oxygen reduction process of 304 stainless steel. This process reduces the cathode reaction and simultaneously inhibits the anodic dissolution reaction of 304 stainless steel, thus achieving 304 stainless steel cathodic protection. Due to the formation of a heterogeneous junction electric field in the Ag/NiS/TiO_2_ nanocomposite, the conductivity potential of the nanocomposite was pulled to a more negative position, which more effectively improved the cathodic protection effect of 304 stainless steel.

## Conclusions

In this paper, nickel sulfide and silver nanoparticles were synthesized on the surface of titanium dioxide nanowires by simple impregnation-deposition and photoreduction methods. A series of studies were conducted on the cathodic protection effect of Ag/NiS/TiO_2_ nanocomposites on 304 stainless steel. Through morphology characterization, composition analysis and light absorption characteristics analysis, the main conclusions are as follows:

1. When the number of nickel sulfide impregnation-deposition cycles was 6, and the silver nitrate photoreduction concentration was 0.1 M, the prepared Ag/NiS/TiO_2_ nanocomposites provided the best cathodic protection for 304 stainless steel. Compared with the saturated calomel electrode, the protective potential reaches − 925 mV, and the protective current reaches 410 µA/cm^2^.
2. A heterojunction electric field is formed at the interface of the Ag/NiS/TiO_2_ nanocomposites, which improves the separation ability of photogenerated electrons and holes. At the same time, it improves the efficiency of light utilization, which makes the absorption range of light expand from the ultraviolet region to the visible region. The nanocomposites will still maintain their initial state after 4 cycles and have good stability.
3. The Ag/NiS/TiO_2_ nanocomposite prepared in the experiment has a uniformly dense surface. Nickel sulfide and silver nanoparticles were uniformly composited on the surface of TiO_2_ nanowires. The composite cobalt ferrite and silver nanoparticles have relatively high purity.
